# Laparoscopic Management of Inguinal Hernia: A Systematic Review and Updated Network Meta-Analysis of Randomized Controlled Trials

**DOI:** 10.7759/cureus.54192

**Published:** 2024-02-14

**Authors:** Hadeel Almutairi, Reem S Alshammari, Majed J Alharbi, Dana M Althobaiti, Raghad S Alghamdi, Samar Alsamiri, Sara W Mawash, Duaa A Ahmed, Abdulrahman A Alamoudi, Fai Y Arif, Fatimah M Albrahim, Mohammed Alfehaid, Haneen W Alanzy

**Affiliations:** 1 Surgery, Unaizah College of Medicine and Medical Sciences, Qassim University, Qassim, SAU; 2 Medicine and Surgery, Northern Border University, Arar, SAU; 3 Medicine, Taibah University, Al Medina, SAU; 4 College of Medicine, Taif University, Taif, SAU; 5 College of Medicine, Umm Al-Qura University, Al-Qunfudhah, SAU; 6 College of Medicine, Imam Mohammad Ibn Saud Islamic University, Riyadh, SAU; 7 College of Medicine, AlMaarefa University, Riyadh, SAU; 8 College of Medicine, King Abdulaziz University, Jeddah, SAU; 9 College of Medicine, Jazan University, Jazan, SAU; 10 College of Medicine, Arabian Gulf University, Alsalmania, BHR; 11 General Surgery, Unaizah College of Medicine and Medical Sciences, Qassim University, Unaizah, SAU; 12 College of Medicine, North Border University, Arar, SAU

**Keywords:** open inguinal hernia repair, direct inguinal hernia, inguinal surgery, inguinal hernia surgery, inguinal hernia repair

## Abstract

Various surgical approaches for inguinal hernia repair have been outlined in medical literature. In most cases, these lesions are repaired by general surgeons. A variety of surgical techniques for the treatment of inguinal hernias have been documented in the medical literature. In 2018, the European Hernia Society (EHS) recommended laparo-endoscopic repair as a preferred approach for adults. This method involves a combination of laparoscopic and endoscopic techniques for hernia repair. The goal of this systematic review is to conduct a thorough examination of the total extraperitoneal vs. transabdominal preperitoneal comparison in inguinal hernia repair, with an emphasis on randomized controlled trials (RCTs). It also intends to conduct a trial sequential analysis (TSA) in order to determine whether more trials and investigations are required or whether there is sufficient evidence to draw a firm conclusion. The study's systematic review and meta-analysis were carried out in accordance with the Preferred Reporting Items for Systematic Reviews and Meta-Analyses (PRISMA) guidelines. We used the PubMed and Google Scholar databases to conduct a thorough web search for articles published between January 2019 and December 2023. The meta-analysis was carried out using Resource Manager Revman version 5.4.1 (Revman International, Inc., New York City, New York). After a review of the studies was done, ten studies were selected to be used in conducting the systematic review and meta-analysis. The recurrence rate of TEP treatment was found to be slightly lower than transabdominal preperitoneal (TAPP). The two techniques did not differ in terms of postoperative complications; however, TEP had a marginally lower rate of postoperative pain. Further, the study revealed that there was a decreased risk of wound infections, seromas, and hematomas with total extraperitoneal (TEP) as opposed to transabdominal preperitoneal (TAPP). TEP also reduced the amount of recovery time needed. After conducting successful hernia treatments, total extraperitoneal and transabdominal preperitoneal both had low rates of complications and recurrence. Based on the information obtained from the study analysis, this meta-analysis provides evidence for the efficacy of TAPP and TEP techniques in the management of inguinal hernias. Though there was a statistically significant difference while applying both methods in the treatment of hernia (p=0.001), TEPs have been shown to have a lower recurrence rate than TAPPs. Similarly, the TEP method has been revealed to have a slight reduction in postoperative pain compared to transabdominal preperitoneal. However, the two techniques have been shown to have no significant difference in postoperative complications. Further, laparoscopic procedures have proved to be a little bit safer and more effective than open procedures. This has been shown by reduced risk of wound infection, hematoma, seroma, and decreased sensibility while using this method. It accelerated the healing process as well. Thus, depending on the needs of the patients and the experience level of the surgeons responsible for the treatments, inguinal hernias can be repaired using either transabdominal preperitoneal or total extraperitoneal techniques since both treatment techniques have generally minimal chance of complications or recurrence as both have proved to safer method.

## Introduction and background

General surgeons are responsible for repairing inguinal hernias, which are said to have an impact on about 15% of adults. Men are about 30% more likely than women to develop an inguinal hernia in their lifetime [1]. The medical literature has outlined various surgical approaches for inguinal hernia repair. Based on the latest European Hernia Society (EHS) guidelines from 2019, the Lichtenstein technique remains a recommended method for open inguinal hernia repair, particularly in specific patient groups. However, there is now a stronger emphasis on the use of minimally invasive procedures, such as laparo-endoscopic techniques, for certain cases due to benefits like lower postoperative pain and reduced chronic pain incidence. These recommendations highlight the importance of surgeon expertise and patient-specific factors in choosing the appropriate surgical approach [1]. 

Minimally invasive surgical techniques are associated with reduced wound-related complications, decreased immediate postoperative pain, expedited recovery allowing for quicker return to work or daily activities, and a lower risk of long-term chronic pain when compared to the traditional open Lichtenstein repair method. On the other hand, transabdominal preperitoneal (TAPP) repair involves the "violation" of the peritoneum, but it offers the benefit of no technical or space limitations and the potential to gain a comprehensive view of the myopectineal orifice, allowing for the detection of unexpected contralateral hernias with thorough preperitoneal dissection [2].

While randomized trials haven't conclusively established one procedure's superiority, some hernia surgeons lean towards the total extraperitoneal (TEP) technique due to its avoidance of complications associated with transabdominal entry. While the European Hernia Society in 2018 does not express a preference between TEP and TAPP, acknowledging both as viable minimally invasive options for hernia repair, TEP's steeper learning curve, attributed to the restricted preperitoneal space, may influence a surgeon's choice towards TAPP, despite both techniques being equally endorsed [3]. 

The aim of this systematic review is to conduct a broad evaluation of the TEP vs. TAPP comparison in inguinal hernia repair, focusing on randomized controlled trials (RCTs). Additionally, it aims to carry out a trial sequential analysis (TSA) to determine whether there is sufficient evidence to draw conclusive conclusions or whether additional trials and investigations are warranted [3] 

## Review

Methods

Literature Search Strategy

The Preferred Reporting Items for Systematic Review and Meta-Analysis (PRISMA) guidelines were followed for this systematic review and meta-analysis. Regardless of time constraints, a thorough electronic search of the PubMed and Google Scholar databases was conducted to locate research articles. Our search strategy included keywords such as surgical techniques for treating inguinal hernias, open or laparoscopic repair, and mesh and hernia operations.

Inclusion and Exclusion Criteria

This review and analysis included all studies published without time constraints, studies that reported different techniques for treating inguinal hernia, all research in English, systematic reviews and cross-sectional studies, meta-analyses, and randomized trials, as well as studies with patients diagnosed with inguinal hernia. Exclusion involved other types of research conducted in other languages, studies involving pediatric patients, those with a high risk of bias or low quality based on evaluations of study design, sample size, data collection and analysis, and other relevant factors, duplicate studies, and those found to have a serious risk of bias.

Selection of Articles and Data Extraction

The main search results were imported into Mendeley (Elsevier, Amsterdam, Netherlands) to remove duplicates. The de-duplicated result was imported into Rayyan and independently screened using the study title and abstract. The complete texts of each study were closely examined, and any discrepancies that arose during the screening phase were resolved by consensus among the authors.

The primary author's name, the year of publication, the country, the type of injury, the sample size, the study design, and the inclusion/exclusion criteria were used to extract data. Social factors such as age, social standing, and treatment method were taken into account during the data extraction process. 

Statistical Analysis

The Resource Manager Revman software version 5.4.1 (Revman International, Inc., New York City, New York) was used for the analysis. The Q-test and the I2 statistic were used to assess the research's heterogeneity. The data were displayed as 95% confidence intervals on forest plots. The odds ratio and standardized mean differences (SMDs) with 95% confidence intervals were calculated for both continuous and categorical outcomes of the forest plots and funnel plots. Risk analysis and funnel plots were used to assess the robustness of the results and the presence of publication bias. A significance level of 0.05 was used for all analyses.

Results

**Figure 1:**
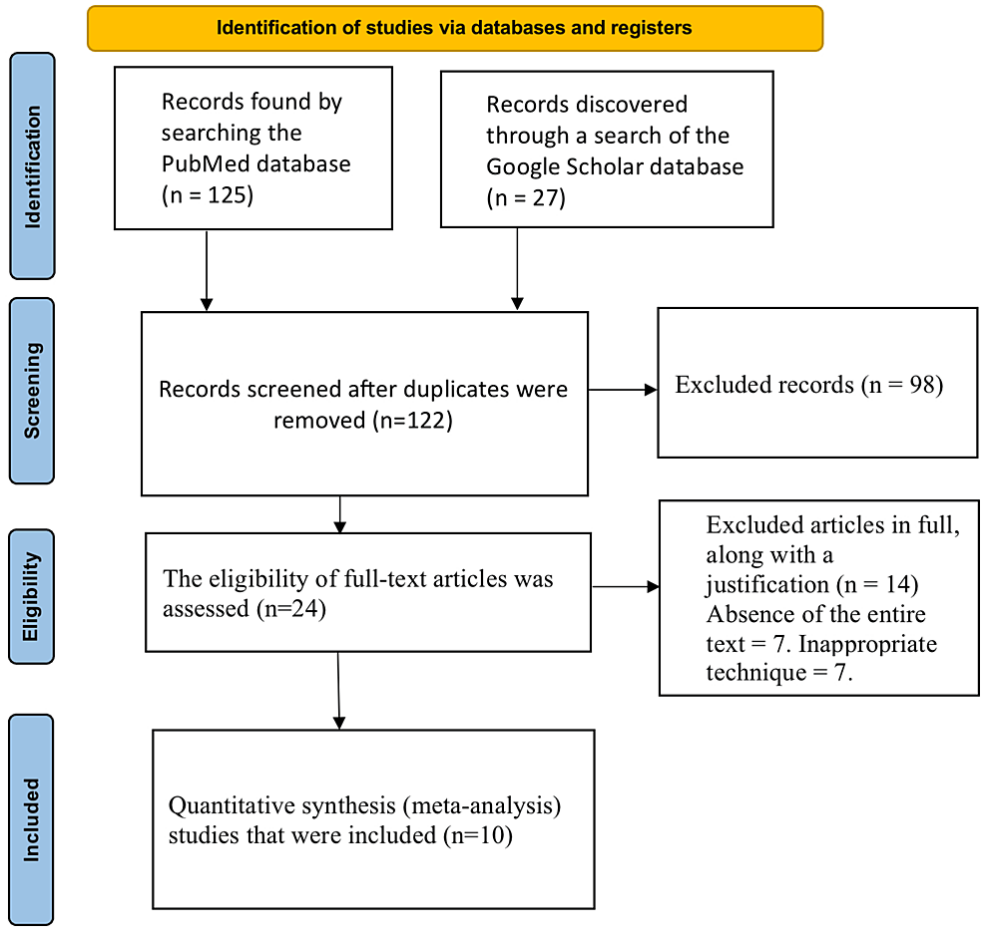
Identification studies via databases and registers

Figure 1 shows a PRISMA diagram of the studies used in this study. A thorough search of several databases yielded 152 studies in total. Following the removal of duplicates, 122 articles were retained for further review. Following the removal of 98 studies during the screening process, 24 articles were retained for eligibility evaluation. Fourteen of these were ruled ineligible (absence of the full text=7; inappropriate technique=7) due to failing to meet the predetermined criteria. In the end, ten studies were selected to be used in conducting the meta-analysis and systematic review.

Characteristics of the Study

Table 1 shows that all 10 studies included were prospective randomized control trials (RCTs) conducted in English. Furthermore, the studies examined were conducted in various regions/countries. The findings show that the studies vary significantly in terms of region, sample size, study design, type of surgery, inclusion, and outcomes. Table 1 shows these variations in each column.

**Table 1:** Descriptive characteristics of the studies

Author	Region	Sample size	Study design	Type of surgeries (if found)	Inclusion	Results/outcome and main findings
Shah et al., 2022 [4]	India	174	Randomized clinical trial	Open/laparoscopic	Unilateral inguinal hernia	Hernia recurrence 2.2%, postoperative complications; chronic groin pain=3.4%; numbness or burning of inguinoscrotal region=4.5%; hematoma=4.5%; seroma=7.9; scrotal swelling/testicular pain=3.4%; spermatic cord edema=2.2; wound infection= 2.2; urinary complaints=6.8%. Postoperative pain 29.5%,
Gavriilidis et al., 2019 [5]		6573	Systematic review, meta-analysis	Open/laparoscopic	Randomized control trials, total extraperitoneal vs. Lichtenstein	Hernia recurrence, laparoscopic=6%; open=4%, postoperative complications hematoma (2.9%-12%) seroma (5%-4.6%) wound infections (1%-2%) vascular injury (1.3%-0.5%) persistent pain (11%-13%) impaired sensibility (6.4%-21%).
Yang et al., 2020 [6]	China	1017	systematic review and meta-analysis	Open/laparoscopic	The study, regardless of blindness, compared laparoscopic and open repair methods for recurrent inguinal hernias. Major related outcomes, such as the rate of recurrence, the length of the operation, and any associated complications, were also reported.	Hernia recurrence, 1.1% to 33.0%, postoperative complications, hematoma, urinary retention, chronic pain. Postoperative pain was common. When compared to the open repair group, the laparoscopic surgery group recovered significantly faster.
Li et al., 2023 [7]	China	180	Randomized clinical trial with prospective blinding	In older patients, propofol anesthesia was induced gradually	Age 60–90 years, elderly patients undergoing laparoscopic inguinal hernia repair, The American Society of Anaesthesiologists defines adequate education and physical status II-III in voluntary participation in this study	Apprehension following surgery Pregabalin premedication may have helped to reduce postoperative pain.
Xie et al., 2019 [8]	China	65	Single-blind, controlled, randomized, prospective study	Paravertebral nerve block and subarachnoid block	Patients are male, 65-89 years old, American Society of Anaesthesiologists I–II, stable blood pressure control, normal heart and lung function, and coordinated treatment.	Discomfort following surgery was clearly noted after 12 and 24 hours.
Srivastava et al., 2023 [9]	India	220	Randomized			One patient in the e- total extraperitoneal group and one patient in the Transabdominal preperitoneal group experienced postoperative complications, including seroma/hematoma and a hernia recurrence. Apprehension following surgery There were only two patients in the Transabdominal preperitoneal group who had persistent thigh pain.
Sevinc et al., 2019 [10]	Turkey	302	Randomized	Open/laparoscopic	Patients who require surgery due to an inguinal hernia	Recurrence of hernia, total extraperitoneal =3.4%. Postoperative complications, odds ratio, occurred in 12 cases (7.7%). We found seroma in 6 (3.8%) of the cases, hematoma in 4 (2.5%) of the cases, and wound infection in 2 (1.2%) of the cases. total extraperitoneal = neumoperitoneum occurred in 6 (4% of the cases). total extraperitoneal =0.7% for postoperative pain odds ratio=1.3%
Koppatz et al., 2019 [11]	Finland	278	Superior prospective single-blind, single-center randomized trial	3rd laparoscopy		Two patients in the 2nd group and three patients in the 3rd group had recurrent inguinal hernias during the one-year follow-up period. Out of all the postoperative Clavien-Dindo classes 1-2, there was only one Clavien-Dindo class 3 complication in the 2nd group. Pain after surgery: 48 patients in the 3rd group experienced mild pain, while three experienced severe pain. In the 2nd group, 34 patients reported mild pain and five reported severe pain.
Zhao et al., 2021 [12]		1379	Meta-analysis and in-depth review	Robotic/laparoscopic		Postoperative pain not significant
Aiolfi et al., 2021 [13]		1359	Systematic review and randomized control trial	Total extra peritoneal / Total abdominal preperitoneal	If there were multiple patients in a duplicate study, only the most comprehensive reports were included for quantitative analysis. English-language articles; two or more publications from the same team, company, or group; the papers with the longest follow-up periods or largest sample sizes; and a randomized controlled trial comparing the surgical outcomes of elective Transabdominal preperitoneal versus total extraperitoneal inguinal hernia mesh repair.	Hernia recurrence has been documented in as many as 2% of cases following total extraperitoneal and Transabdominal preperitoneal repair. Postoperative complications, including wound-related complications, were discovered to have comparable rates of hematoma, seroma, and wound infection. The length of hospitalization and the risk-to-benefit ratio. Up to 3% of patients have reported chronic pain after minimally invasive surgery.

Risk of Bias Analysis

**Figure 2:**
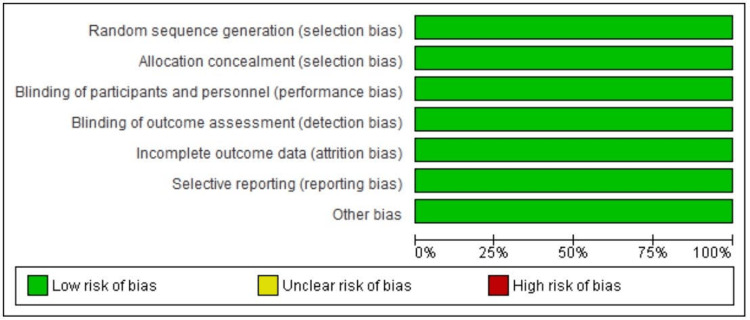
Risk of bias analysis

The study's findings show that none of the bias risks examined, such as incomplete outcome data, selective reporting, blinding of participants and staff, random sequence generation, allocation concealment, and other types of biases were revealed by the studies included in the systematic and meta-analysis. Figure 2 depicts the results.

Forest Plot for Recovery Time

**Figure 3:**
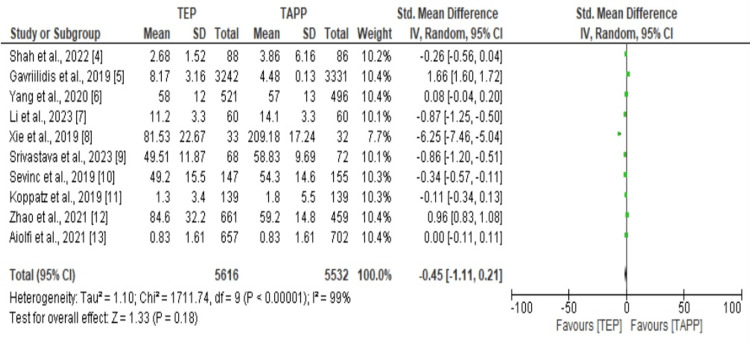
The forest plot for recovery time.

The meta-analysis included ten studies with a total sample size of 11,547 participants. The ten studies' sample sizes ranged from 65 to 5673. However, some participants dropped from the study, thus leading to the sample size being reduced to 11,148. The forest plot results in Figure 3 below show that there is significant heterogeneity across all studies. It's interesting to note that each study had the appropriate weighting (SMD -0.45, 95% CI -1.11 to 0.11; I2 = 99%). Even though the TAPP method requires a slightly shorter recovery time than the TEP treatment method, there is no discernible difference in recovery time between the two techniques.

Forest Plot for Postoperative Complications of the Used Studies

**Figure 4:**
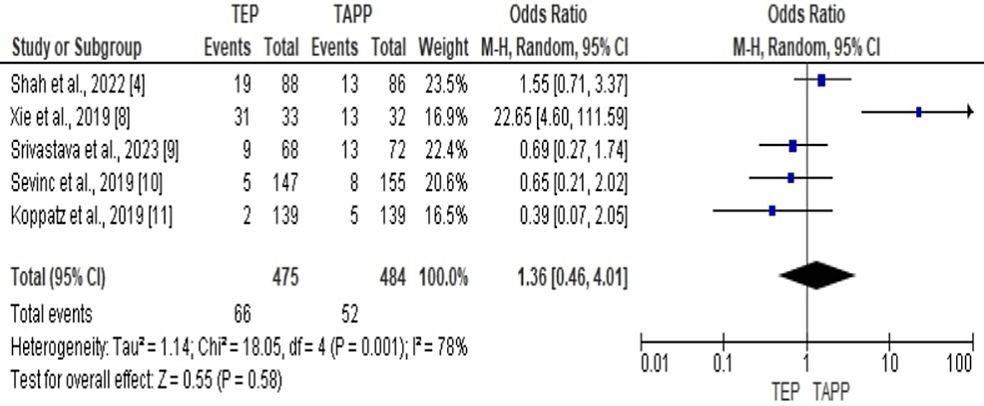
The forest plot for postoperative complications of the used studies

The forest plot results in Figure 4 show statistically significant heterogeneity across the studies used in comparing the postoperative complication, with an I2 value of 78% RE 1.36, 95% CI 0.46 to 4.01. However, the overall effect of the two treatment methods was not statistically significantly different. 

Funnel Plot

**Figure 5:**
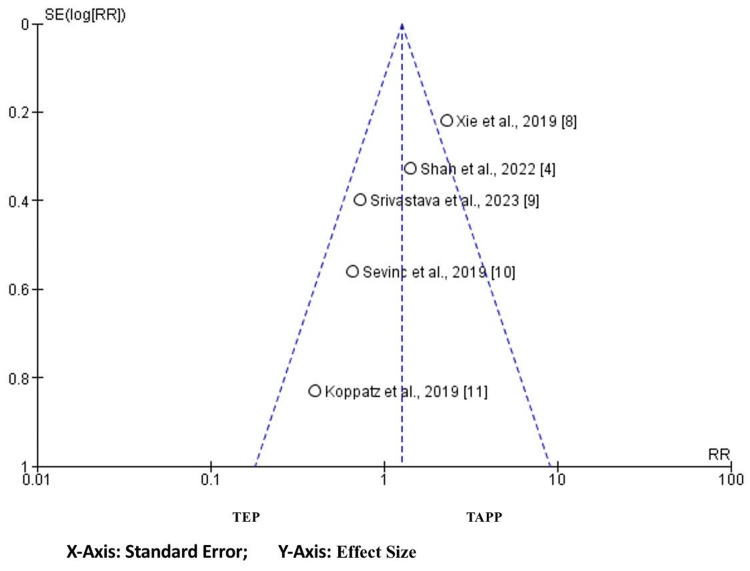
Funnel plot depicting publication bias.

An asymmetrical funnel was portrayed by funnel plot analysis. These indicate there are chances of bias on both sides and mostly to the left; the results are depicted in Figure 5.

Discussion

The primary aim of this systematic review and meta-analysis was to conduct a comprehensive assessment of the TEP vs. TAPP comparison in inguinal hernia repair, with a focus on randomized controlled trials (RCTs). The aim of the study was to conduct a trial sequential analysis (TSA) to ascertain the necessity of additional trials and investigations or if the available data sufficed for making informed decisions. According to the study findings, the difference in the treatment methods was very minimal. For TEP, hernia recurrence was 2.2%, postoperative complications for chronic groin pain were 3.4%, inguinoscrotal numbness or burning was 4.5%, hematoma was 4.5%, seroma was 7.9%, scrotal swelling/testicular pain was 3.4%, spermatic cord edema was 2.2%, and wound infection was 2.2%. The study further revealed that urinary complaints were reported by 6.8% of patients, and postoperative pain was reported by 29.5%, which was a little bit lower compared to TAPP, though the difference was insignificant (p<0.05) [4].

In addition, in a study by Gavriilidis et al., the comparison between the two methods on postoperative complications was revealed to be hematoma (2.9% vs. 12%), seroma (5% vs. 4.6%), wound infections (1% vs. 2%), vascular injury (1.3% vs. 0.5%), persistent pain (11% vs. 13%), impaired sensibility (6.4% vs. 21%). This implies that laparoscopic surgery was more efficient than open repair in most of the cases [5].

Further, a study by Yang et al. revealed that hernia recurrence for TEP vs TAPP was 1.1% and 3.3%, respectively. However, there were no significant differences in both treatment methods for postoperative complications, hematoma, urinary retention, chronic pain, and postoperative pain. Recovery time of the laparoscopic group was significantly shorter than that of the open repair group [6]. A study by Li et al. noted that the postoperative pain and the use of pregabalin as premedication may be responsible for attenuating the postoperative pain [7]. On the other hand, the study noted that in the laparoscopic treatment method, the postoperative pain appeared for 12 hours, while in the open repair method, it appeared for 24 hours [8].

In each method of treatment, there were no significant differences in terms of hernia recurrence; one patient was noted to have hernia recurrence in the TEP group and one patient in the TAPP group. Similarly, postoperative complications, seroma/hematoma, and postoperative pain in chronic thigh pain occurred only in two patients in the TAPP group, which was not statistically different from the TEP method [9]. Hernia recurrence is 3.4% after total extraperitoneal (TEP) surgery, but it is slightly higher after open hernia repair (OPR), at 5.2%. This suggests that the TEP strategy may be slightly more effective than OPR in preventing hernia recurrence. Postoperative complications were also 7.7% higher in the open repair group (0.7%) compared to the TEP group. The three most typical side effects were wound infection, seroma, and hemostasis. From this, it can be concluded that the TEP technique may result in fewer surgical complications than open repair. Some of the unique and specific problems associated with each technique found that during the TEP procedure, pneumoperitoneum formation occurred in 4% of cases. Although it usually resolves on its own and doesn't require further care, this is a well-known side effect of the TEP technique. Furthermore, compared to the open repair group, the TEP group reported 0.7% less pain after surgery [10].

In the TEP group, there was just one instance of a more severe postoperative complication, as was the case of the TAPP group. This implies that there is a decreased chance of complications with both the TEP and TAPP methods. Both treatment methods were shown to cause postoperative pain in the patients. The patients treated using the TAPP method were shown to have a higher level of severe pain than the patients treated using the TEP method [11].

Nevertheless, the study found that there is no statistically significant in regard to postoperative pain when it comes to hernia repair with the TEP or TAPP technique. Hernia recurrence was reported to be 2% when utilizing both approaches in the treatment [12]. Additionally, it was found that there is no statistically significant difference between the TEP and TAPP approaches in terms of the risk of infection, seroma, and hematoma after surgery. This implies that both methods have minimal distinction in terms of efficiency and safety of patients in treatments. Nevertheless, following a minimally invasive hernia repair procedure, up to 3% of patients experience chronic pain. This suggests that while moderate pain following surgery is possible, chronic pain lasting a lifetime is also possible [13].

## Conclusions

Based on the information obtained from the study analysis, this meta-analysis provides evidence for the efficacy of TAPP and TEP techniques in the management of inguinal hernias. Though there were statistically significant differences while applying both methods in the treatment of hernia (p=0.001), TEPs were shown to have a lower recurrence rate than TAPPs. Similarly, the TEP method was revealed to have a slight reduction in postoperative pain than TAPP. However, the two techniques were shown to have no significant difference in postoperative complications. Further, laparoscopic procedures have proved to be a little bit safer and more effective than open procedures. This has been shown by reduced risk of wound infection, hematoma, seroma, and decreased sensibility while using this method. It accelerated the healing process as well. Thus, depending on the needs of the patients and the experience level of the surgeons responsible for the treatments, inguinal hernias can be repaired using either TAPP or TEP techniques since both treatment techniques have generally minimal chance of complications or recurrence as both have proved to be safe methods.
